# Time to reality check the promises of machine learning-powered precision medicine

**DOI:** 10.1016/S2589-7500(20)30200-4

**Published:** 2020-09-16

**Authors:** Jack Wilkinson, Kellyn F Arnold, Eleanor J Murray, Maarten van Smeden, Kareem Carr, Rachel Sippy, Marc de Kamps, Andrew Beam, Stefan Konigorski, Christoph Lippert, Mark S Gilthorpe, Peter W G Tennant

**Affiliations:** Centre for Biostatistics, Manchester Academic Health Science Centre, Division of Population Health, Health Services Research and Primary Care, University of Manchester, Manchester, UK; Leeds Institute for Data Analytics, University of Leeds, Leeds, UK; Faculty of Medicine and Health, University of Leeds, Leeds, UK; Department of Epidemiology, Boston University School of Public Health, Boston, MA, USA; Department of Clinical Epidemiology, Leiden University Medical Center, Leiden, Netherlands; Department of Biostatistics, Harvard TH Chan School of Public Health, Boston, MA, USA; Institute for Global Health and Translational Science, SUNY Upstate Medical University, Syracuse, NY, USA; Department of Geography, University of Florida, Gainesville, FL, USA; Emerging Pathogens Institute, University of Florida, Gainesville, FL, USA; Leeds Institute for Data Analytics, University of Leeds, Leeds, UK; School of Computing, University of Leeds, Leeds, UK; Department of Epidemiology, Boston University School of Public Health, Boston, MA, USA; Digital Health & Machine Learning Research Group, Hasso Plattner Institut for Digital Engineering, Potsdam, Germany; Hasso Plattner Institute for Digital Health at Mount Sinai, Icahn School of Medicine at Mount Sinai, New York, NY, USA; Digital Health & Machine Learning Research Group, Hasso Plattner Institut for Digital Engineering, Potsdam, Germany; Hasso Plattner Institute for Digital Health at Mount Sinai, Icahn School of Medicine at Mount Sinai, New York, NY, USA; Leeds Institute for Data Analytics, University of Leeds, Leeds, UK; Faculty of Medicine and Health, University of Leeds, Leeds, UK; Alan Turing Institute, London, UK; Leeds Institute for Data Analytics, University of Leeds, Leeds, UK; Faculty of Medicine and Health, University of Leeds, Leeds, UK; Alan Turing Institute, London, UK

## Abstract

Machine learning methods, combined with large electronic health databases, could enable a personalised approach to medicine through improved diagnosis and prediction of individual responses to therapies. If successful, this strategy would represent a revolution in clinical research and practice. However, although the vision of individually tailored medicine is alluring, there is a need to distinguish genuine potential from hype. We argue that the goal of personalised medical care faces serious challenges, many of which cannot be addressed through algorithmic complexity, and call for collaboration between traditional methodologists and experts in medical machine learning to avoid extensive research waste.

## Introduction

Proponents of precision medicine make a compelling pitch: traditional approaches to health science have focused too much on comparing effectiveness in the average person and too little on the needs of actual individuals.^1,2^ The blame could lie with outdated statistical and epidemiological tools, which might offer decreasing relevance to the needs of contemporary clinical decision making.^3^ The proposed solution speaks to the zeitgeist: our newfound abundance of detailed and accessible longitudinal data on individuals combined with the practical realisation of various flexible machine learning approaches offer an exciting chance for revolution.^2^ At the apex sits the dream of precision medicine crafted by machine learning, a new framework that promises to revolutionise how we identify the best therapy for each person as an individual, while automating everyday tasks like diagnosis and prognostication with unprecedented accuracy.^4^

But how realistic are these claims? And when, if ever, can we expect them to be routinely realised? We consider the evidence underlying two of the most common claims about the potential of machine learning-powered precision medicine and call for a reality check of expectations.

## Claim 1: machine learning will enable automated diagnoses with unprecedented accuracy

Machine learning is often heralded by health and medical commentators as a powerful prediction tool that will revolutionise disease screening and diagnosis. The inherent flexibility and scope for automation makes machine learning well suited to examining complex high-dimensional data (ie, with many variables or features) that would be challenging to model using conventional approaches. Such strategies have enabled the development of several innovative diagnostic algorithms—for example, to identify patients most in need of intervention from knee MRI,^5^ to detect cardiac arrhythmias from electrocardiograms,^6^ and to diagnose pneumonia from chest x-rays.^7^

Given such innovation, it is hard to dispute the revolutionary potential of machine learning for improving clinical diagnostics. However, acknowledging potential is a poor substitute for robust scientific evidence of actual benefit, and here research is lacking.^8^ Although news media is filled with enthusiastic stories about novel machine learning applications,^9–12^ a systematic review comparing the performance of deep learning versus health professional assessment in diagnosis of various diseases from medical images makes for sobering reading.^13^ Only 20 (24%) of the 82 studies identified evaluated the performance of their algorithm in an external cohort, and only 14 (17%) studies compared this out-of-sample performance with that of health professionals. This number is alarmingly small, especially given that many of the studies were flawed. The authors found that reporting standards were typically poor, internal validation was weak and, perhaps most worryingly, model performance was often evaluated under unrealistic conditions that had little relevance to routine clinical practice.^13^ For example, there is little use comparing the performance of a machine learning algorithm with health professional judgment for making diagnoses from medical images without providing further contextual information about the patient, as this would never happen in practice.^14^ A more recent systematic review of studies comparing deep learning with clinical judgement corroborated the widespread issues with poor study design and reporting, but identified some well designed randomised clinical trials that evaluated the technology.^15^

Emphasis on predictive performance over clinical utility is not unusual. The ability of machine learning to process high-dimensional data, for example, appears to be distracting from the often greater benefits of simple clinical variables. Volkmann and colleagues^16^ showed how predictions based on large dimensional omics data can easily be improved by including more common clinical information. Indeed, the added value of omics data for clinical prediction can be marginal once all relevant clinical variables are included. There is also an increasing focus on classifying patients into simple categories (eg, with and without the disease) rather than predicting a continuum of risk. This trend is an unfortunate departure from the supposed aim of increasing individual relevance and is particularly puzzling because it is not a requirement of most machine learning methods.

In terms of using machine learning to automate diagnoses, this remains more of a potential promise than a proven product, although notable exceptions exist, such as an automated system for detection of diabetic retinopathy.^17^ At present, the benefits of most proposals to automate clinical diagnoses with machine learning are unknown because they have not been meaningfully assessed. Novel machine learning studies are not unusual in this regard. Clinical journals are flooded with traditional prognostic models that were neither developed nor evaluated using appropriate methods.^18^ However, since few of these models ever end up being used, they are arguably benign. By contrast, there is tangible concern that the scale of enthusiasm around machine learning means substandard models might get unduly adopted by a clinical audience that is not equipped to assess them.

Regardless, the performance and utility of a machine learning algorithm is highly dependent on the quality and relevance of the data on which it is trained. Training in image recognition requires large numbers of images to be scrutinised and annotated by human experts, a burdensome task that itself carries a risk of error, highlighting the need for methods to enable medical experts to create high-quality annotations at scale. Furthermore, algorithms often perform badly and require retraining when introduced into environments that were not represented in their training data, as highlighted by the poor performance of a Google algorithm for detecting diabetic retinopathy from retinal images when deployed in poorly-lit eye clinics in Thailand.^19^

## Claim 2: machine learning-powered precision medicine will enable identification of the best therapies for individuals rather than groups

The potential to revolutionise the individual tailoring of medical treatments is one of the most widely discussed and appealing promises of machine learning-powered precision medicine.^20^ Unfortunately, this promise is probably also the least likely to ever be fully achieved, for two fundamental epistemological reasons.

First, machine learning approaches are not (currently) able to identify cause and effect, because causal inference is fundamentally impossible to achieve without making assumptions.^21,22^ Causal inference requires the following three central assumptions: a clearly specified causal question, that the causal effect of interest can be identified, and that all treatment options of interest can be observed among all groups of interest. Several of these assumptions can be satisfied through study design or external contextual knowledge (ie, human input or true artificial intelligence), but none can be discovered solely from observational data. Since causal inference is necessary to find out what works and by how much, this issue poses a considerable barrier to the promise of ever being able to identify the best treatment for an individual person by machine learning.

This problem does not simply reflect the adage that correlation does not imply causation, nor the widely held belief that causal inference can only be achieved in experimental data. A suite of methodological approaches are available to aid estimation of causal effects in non-experimental data,^23,24^ such as electronic health records, which potentially offer more diverse and relevant samples than clinical trials (although representative samples might not be necessary to achieve representative results^25^). The limitation arises because almost all machine learning algorithms have been designed to make predictions (eg, to most accurately predict or classify those with a particular trait or prognosis) and this is fundamentally distinct from causal explanation and causal effect estimation.^26,27^

To estimate a causal effect, we instead need to estimate not just what is most likely to happen (ie, prediction) but what would most likely have happened if things had been different (ie, counterfactual prediction^27^). Accordingly, the prospect of automated causal inference in observational data remains beyond reach. Instead, causal inference requires external contextual information about the meaning of and relationships between the relevant variables in a given context.^28^ Machine learning cannot learn these things from a dataset because they are not necessarily there to learn—the data only include what has happened, not what might have happened if things had been different. Hence, data-driven prediction models cannot help us to determine the effect of different exposures and treatments on different outcomes, and in turn cannot deliver the promise of identifying the best treatment approach for specific individuals.^28^ Simply increasing the sample size (ie, collecting big data) or increasing algorithmic complexity does not help to resolve this fundamental epistemological mismatch of aims. Further tensions arise between adopting a data-driven approach and the preference towards pre-specification of analyses before data collection in clinical trials.

However, that machine learning cannot yet—if ever—conduct causal inference is arguably less of a barrier to achieving individual-level predictions than the second more fundamental problem that the majority of health states and events are so complex that we can only understand them probabilistically, and chance can never be predicted at the individual level. Indeed, although statistics—and hence machine learning—is excellent at helping us to understand and compare probabilities between groups, it is fundamentally unable to tell us what will happen to an individual. The power of statistics is precisely that it can describe and predict partly random events over large numbers of people. But no matter how accurately statistics can do this, it can never tell us with certainty what the next event will be.

For example, consider rolling two (fair) dice and counting the total score. Although we know what is most likely (seven), and we know with 100% certainty the probability of all other scores (eg, 1 in 32 for a double six), we are still no closer to knowing what the next roll will actually be. Likewise, even the most sophisticated causal inference methods cannot determine with certainty the effect of an individual variable in an individual person (eg, the effect of systolic blood pressure on the risk of stroke). This issue is not resolvable by simply collecting more data or building increasingly complex models, because it stems from limitations of our understanding of physical and biological processes. Since most health processes are effectively probabilistic, we must accept that individual outcomes will always be subject to chance, no matter how precisely we can describe these for groups of similar patients.

## Pragmatic routes to personalisation

The scale and substance of these barriers leads to a larger question: is precision medicine itself an epistemological dead end? To answer, we must step back from the headline promises and revisit the core principles. The first requirement for precision medicine is that treatments have different effects in different people and the second is that this variation can, at least partly, be characterised and predicted.^29^ Where the cause of such variation can be easily identified and targeted, such as with a disease that has a dominant genetic component, this is potentially realistic; indeed this is exactly where precision medicine has found its success.^30^ However, in most cases, characterising and predicting treatment responses is undermined for the very reasons we seek to use precision medicine—health is complex—and understanding the determinants of variation in response is a formidable challenge. Indeed, for most health states and events, we remain unable to achieve the ostensibly much easier task of improving the average response in a group.

One of the biggest obstacles to precision medicine comes from our poor collective understanding of the nature of variation and the types of study needed to reveal it. Disease symptoms and the apparent treatment response can vary substantially within the same individual over time.^29^ Traditional clinical studies are woefully ill-equipped to identify who will respond consistently well, or indeed whether anyone will respond consistently at all. Even with more intensive study designs, such as repeated crossover studies or N-of-1 trials, it might not be possible to differentiate within-individual and between-individual variation without strong assumptions that are only plausible in specific circumstances (eg, where the symptoms and participant circumstances are stable over time and the treatments have no long-term effects).^29,31^

A more pragmatic route towards greater personalisation might be to shift the aim towards stratified medicine (ie, identifying and predicting subgroups with a better and worse response). Although somewhat more modest in ambition, this strategy would be compatible with our existing statistical epistemology and could still provide a genuine revolution. However, this route remains much more complex than simply applying machine learning approaches to experimental or observational datasets, because of the fundamental challenge of differentiating true signal from noise. For example, response variation is typically explored by simple subgroup analyses of specific predefined variables (eg, sex, age, race, or ethnicity). These analyses are usually exploratory, because even if there are sound expectations of treatment variation, experiments tend to be restricted to focus on the responsive group. However, doing multiple statistical tests for modest effects in relatively small samples is a recipe for disaster, including mistaking noise for signal, exaggerating trivial effects, and (ironically) overlooking true effects.^32–34^ Anything that is identified of course needs validation in subsequent experiments,^35^ which are rare and seldom confirm the initial suspicions.^33,36^

## Conclusion

Both machine learning and precision medicine are genuine innovations and will undoubtedly lead to some great scientific successes. However, these benefits currently fall short of the hype and expectation that has grown around them. Such a disconnect is not benign and risks overlooking rigour for rhetoric and inflating a bubble of hope that could irretrievably damage public trust when it bursts. Such mistakes and harm are inevitable if machine learning is mistakenly thought to bypass the need for genuine scientific expertise and scrutiny. There is no question that the appearance of big data and machine learning offer an exciting chance for revolution, but revolutions demand greater scrutiny, not less. This scrutiny should involve a reality check on the promises of machine learning-powered precision medicine and an enhanced focus on the core principles of good data science—trained experts in study design, data system design, and causal inference asking clear and important questions using high-quality data.
